# Significance of delayed primary excision in localized nonmetastatic adult head and neck rhabdomyosarcoma

**DOI:** 10.1002/cam4.855

**Published:** 2016-08-26

**Authors:** Kenya Kobayashi, Fumihiko Matsumoto, Makoto Kodaira, Taisuke Mori, Naoya Murakami, Akihiko Yoshida, Daisuke Maki, Masanori Teshima, Masahiko Fukasawa, Jun Itami, Masahiro Asai, Seiichi Yoshimoto

**Affiliations:** ^1^ Department of Head and Neck OncologyNational Cancer Center HospitalTokyoJapan; ^2^ Department of Medical Oncology and Breast OncologyNational Cancer Center HospitalTokyoJapan; ^3^ Department of PathologyNational Cancer Center HospitalTokyoJapan; ^4^ Department of Radiation OncologyNational Cancer Center HospitalTokyoJapan; ^5^ Department of OtolaryngologyKamagaya General HospitalTokyoJapan

**Keywords:** Adult rhabdomyosarcoma, delayed primary excision, head and neck sarcoma, induction chemotherapy, surgery

## Abstract

Adult rhabdomyosarcoma (RMS) is a highly aggressive tumor. Multidisciplinary treatment is important. However, the role of surgery is controversial. The purpose of this study was to reveal the role of a delayed primary excision (DPE) after induction chemotherapy (IC) in localized nonmetastatic adult head and neck RMS. We retrospectively reviewed 24 adult head and neck RMS. Treatment was classified into the following two groups: the DPE group, who received IC followed by surgery, postoperative radiotherapy, and adjuvant chemotherapy (17 patients); the chemoradiotherapy (CRT) group, who received IC followed by chemoradiotherapy (seven patients). We analyzed the efficacy of IC, local control rate (LCR), and overall survival (OS). In the DPE group, 10 patients (59%) underwent complete surgical resection. In the evaluation of the surgical specimens, 14 patients (82%) had residual viable tumors after IC. The response to IC was significantly associated with the 3‐year LCR (CR/PR vs. SD/PD: 100% vs. 33%, *P *=* *0.0014). In patients with good response to chemotherapy, the DPE group had a significantly better 3‐year LCR compared with that of the CRT group (DPE group vs. CRT group, 100% vs. 44%, *P *=* *0.018). However, the treatment modalities were not associated with OS (DPE group vs. CRT group, 65% vs. 57%: *P *=* *0.98). The recurrence patterns differed according to treatments, and distant metastases were more frequent in the DPE group. DPE may impact local control of localized nonmetastatic adult head and neck RMS. Poor response to IC is a risk factor for local recurrence.

## Introduction

The head and neck is a common site for rhabdomyosarcoma (RMS), and 34% of all pediatric patient tumors occur in the head and neck 1. These tumors are divided into three categories: parameningeal, orbital, and nonorbital nonparameningeal. Almost all cases develop at an early age, so adult patients are rare. In general, RMSs in adults are highly aggressive and have a poorer prognosis than in pediatric patients 2, 3.

In management, there is no doubt about the importance of multidisciplinary treatment. However, for pediatric head and neck RMS, the role of surgery is controversial because the tumors are typically very sensitive to chemotherapy and radiotherapy 4 and complete surgical resection can be difficult because of anatomical, cosmetic, or functional reasons. Moreover, the significance of surgery after induction chemotherapy (IC), that is, delayed primary excision (DPE) is unknown. The aim of this study was to reveal the role of surgery, with a particular focus on DPE, in the treatment of localized nonmetastatic adult head and neck RMS.

## Materials and Methods

### Study population

From January 2003 to December 2013, 37 adult head and neck RMSs were treated at the National Cancer Center Hospital of Japan. Of these 37 patients, three patients directly underwent surgery because of small primary lesion, four patients refused surgery at their own will, and six patients underwent palliative therapy because of their general condition or massive distant metastasis. These 13 patients were excluded from the analysis. The remaining 24 patients were considered eligible for this retrospective analysis. The patient characteristics are shown in Table 1.

**Table 1:** Patient characteristics

Characteristic	DPE group	CRT group	Total
Sex			
Male/Female, No.	8/9	3/4	11/13
Median age, years (range)	26 (19–60)	25 (19–32)	26 (19–60)
Primary tumor site, No.			
Paranasal sinus	6	5	11
Nasal cavity	6	1	7
Parapharyngeal space	2	0	2
Oral cavity	1	1	2
Nasopharynx	1	0	1
Others	1	0	1
T classification, No.			
T1a/T1b/T2a/T2b	5/2/3/7	3/0/0/4	8/2/3/11
N classification, No.			
N0/N1	11/6	3/4	14/10
M classification, No.			
M0/M1	17/0	2/5	19/5
SPIO presurgical staging, No.			
I/II/III/IV	1/4/11/1	0/0/2/5	1/4/13/6
Resectability in initial image study, No.			
Resectable/Unresectable	17/0	4/3	21/3
Histopathological type, No.			
Embryonal/Alveolar/Others	3/11/3	1/6/0	4/17/3

DPE, delayed primary excision; CRT, chemoradiotherapy; SPIO, International Society of Pediatric Oncology; No., Number of patients.

The study population consisted of 11 men and 13 women with an age distribution of 19–60 years (median age: 26 years). All patients were older than 18 years of age. The median follow‐up period was 36 months (range: 10–80 months). All patients had been previously untreated.

The 4th edition of the World Health Organization (WHO) classification was used for histologic classification. The 7th edition of the TNM classification from the International Union against Cancer (UICC) and the American Joint Committee (AJCC) on Cancer, the International Society of Pediatric Oncology (SPIO) presurgical staging classification, and the North America Intergroup Rhabdomyosarcoma Study Group (IRSG) postsurgical grouping classification were adopted for clinical staging. The FOXO1 rearrangement status was analyzed using fluorescence in situ hybridization to support the tumor subtyping in select cases. Response evaluation after IC was defined as follows. Complete response (CR) was defined as the complete disappearance of disease, good partial response (good PR) was defined as a tumor reduction of >60% but not CR, and minor partial response (minor PR) was defined as a tumor reduction of >30% but not good PR. No response or a reduction of <30% was classified as stable disease (SD), and an increase in tumor size or the detection of new lesions was classified as the progression of disease (PD).

### Treatment strategy

**Figure 1:**
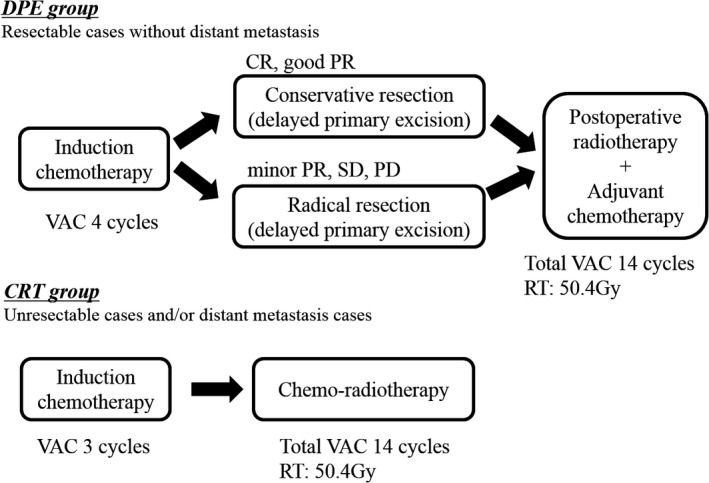
Main Treatment Strategies. The main treatment strategies for the two groups are shown in Figure 1.

Figure 1 shows the main treatment strategies used in this study. Because most adult head and neck RMSs are locally advanced and are sensitive to chemotherapy, we typically performed DPE without initial radical resection.

The treatment was mainly classified into two groups. If the primary tumor was resectable, IC followed by surgery, postoperative radiotherapy, and adjuvant chemotherapy was performed (DPE group). If the primary tumor was unresectable or distant metastases were present, IC followed by chemoradiotherapy and adjuvant chemotherapy without surgery was performed (CRT group). The definition of unresectability was invasion of cavernous sinus, carotid artery, wide dura, and cerebrum.

In the DPE group, the standard induction regimen was VAC (Vincristine 1.5 mg/m^2^, Actinomycin‐D 0.045 mg/kg, and Cyclophosphamide 2200 mg/m^2^) chemotherapy. After four cycles of chemotherapy, we performed a reevaluation of the tumor. If a complete surgical resection was possible after IC, we performed surgery.

In principle, we performed radical resections based on the initial imaging studies. However, in cases with good response to IC (more than good PR), we performed a conservative resection based on the repeat imaging studies obtained after chemotherapy.

After surgery, we promptly started postoperative radiotherapy and adjuvant chemotherapy within a median of 24 days. The patients received 50.4 Gy in 1.8 Gy fractions to the primary tumors as per IRSG postsurgical grouping classification Group III. For systemic therapy, we administered total 14 cycles of adjuvant VAC chemotherapy (Vincristine 1.5 mg/m^2^, Actinomycin‐D 0.045 mg/kg, and Cyclophosphamide 1200 mg/m^2^) after local treatment.

In the chemoradiotherapy (CRT) group, we performed chemoradiotherapy without a surgical resection after three cycles of IC. The standard regimen was the same, 14 cycles of VAC chemotherapy. For radiotherapy, the patients received 50.4 Gy in 1.8 Gy fractions to the primary tumors.

To the case of local recurrence, we performed radical salvage surgery. The second‐line chemotherapy was performed in the cases of unresectable or distant recurrences. In total, the DPE group contained 17 patients and CRT group contained seven patients. In CRT group, three patients were unresectable in initial image studies.

The study was approved by our institutional ethics committee (2010‐077) and conducted in accordance with the Declaration of Helsinki.

### Statistical analysis

We retrospectively analyzed the recurrence pattern, risk factors for local recurrence, 3‐year local control rate (LCR), and overall survival (OS). LCR and OS were calculated using the Kaplan–Meier method, and the differences were analyzed using log‐rank tests. A value of *P *<* *0.05 was considered statistically significant. The analyses were conducted using Statmate Version 2 (GraphPad, La Jolla, CA).

## Results

### Clinicopathological response to induction chemotherapy and surgery

**Figure 2:**
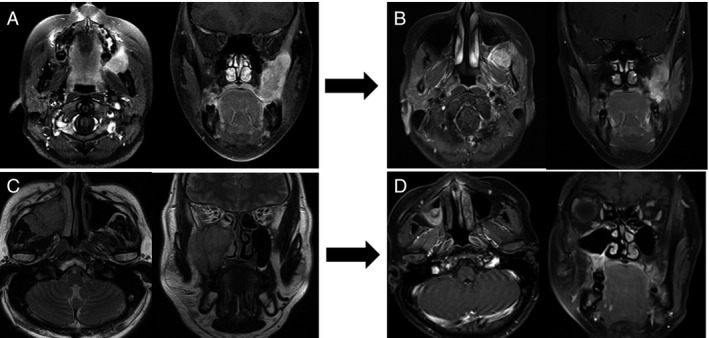
Case Presentation. A 21‐year‐old woman with a parapharyngeal primary tumor (A). She underwent four cycles of VAC chemotherapy, and the tumor response was SD (B). A radical resection was performed in this case. A 44‐year‐old man with a maxillary sinus primary tumor (C). He underwent four cycles of VAC chemotherapy and achieved CR (D). A conservative resection was performed in this case. Local Control Rate and Overall Survival by the DPE and CRT groups. CRT, chemoradiotherapy; DPE, delayed primary excision; VAC, Vincristine.

For the DPE group, the clinical response after IC was SD/PD in four patients, PR in seven patients (good PR, three patients; minor PR, four patients), and CR in six patients. Radical resection was performed for four patients of PD/SD and four patients of the minor PR. On the other hand, conservative resection was performed for three patients of good PR and six patients of the CR. The details for the surgeries are shown in Table 2, and the cases are presented in Figure 2.

**Table 2:** Response to induction chemotherapy and surgery

Response	No. of Pt	Extent of resection	Detail of surgery (No. of Pt)
PD/SD	4	Radical	SB (2), RwF (2)
Minor PR	4	Radical	SB (1), RwF (3)
Good PR	3	Conservative	PM (3)
CR	6	Conservative	PM (4), ND (2)

No. of Pt, Number of patients; PD, progression of disease; SD, stable disease; PR, partial response; CR, complete response; SB, skull base surgery; RwF, resection with free flap reconstruction; PM, partial maxillectomy; ND, neck dissection.

In the evaluation of the surgical specimens, three patients were pathologically found to be CR. However, the remaining 14 patients had residual viable tumors. Moreover, in the six patients of clinical CR after IC, there were only two patients with pathological CR.

On the other hand, in the CRT group, the response was SD/PD in one patient, PR in three patients (good PR, three patients), and CR in three patients after IC.

### Overall outcome

**Figure 3:**
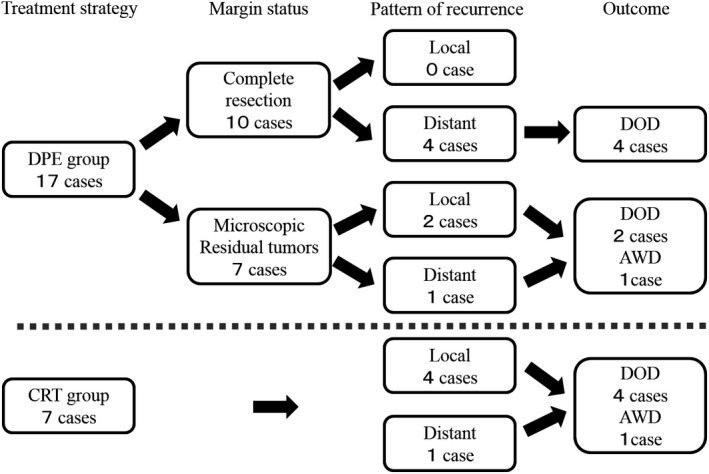
Treatment Outcome. The overall outcomes for the two groups are presented. DOD, die of disease; AWD, alive with disease.

The overall outcomes are presented in Figure 3. Of the 17 patients treated with surgery (DPE group), 10 patients underwent a complete resection with negative surgical margins without any local recurrence. On the other hand, the remaining seven patients underwent a gross total resection that left microscopic residual tumors; there were only two patients who had local recurrences. In both patients, the response to the IC was PD. Even if local control was achieved, distant metastases were detected in five patients. Salvage treatments for these recurrences were difficult.

Of the seven patients treated using CRT, local recurrence was detected in four patients and distant recurrence was identified in one patient. Salvage treatments for these recurrences were difficult. The recurrence pattern differed between the two groups because distant metastases were more common in the DPE group, whereas local recurrences were more common in the CRT group.

### Risk factors for local recurrence

The response to IC was significantly associated with the 3‐year LCR (CR/PR vs. SD/PD: 100% vs. 33%, *P *=* *0.0014). In the patients who were good response to IC (CR/PR), there were no local recurrences, even if microscopic residual tumors were present. Having a large primary tumor, advanced stage disease, embryonal pathological type, viable tumor in DPE specimens, and parameningeal primary lesion were associated with poorer 3‐year LCR, but these results were not statistically significant (Table 3).

**Table 3:** Risk factors of local recurrence in the DPE group

Risk factor	3‐year LCR (%)	*P* value
Tumor size		
<5 cm	100	0.12
>5 cm	71	
Stage		
I or II	100	0.36
III or IV	82	
Age		
<20	86	0.89
>20	88	
Pathological type		
Embryonal	50	0.20
Alveolar/Others	92	
Primary location		
Parameningeal	85	0.57
Others	100	
Extent of residual tumor		
Microscopic	71	0.11
Complete resection	100	
Extent of surgery		
Conservative surgery	100	0.11
Radical surgery	71	
Viable tumor in DPE specimens		
Negative	100	0.53
Positive	85	
Response to IC		
PR/CR	100	0.0014
PD/SD	33	

DPE, delayed, delayed primary excision; IC, induction chemotherapy; PD, progression of disease; SD, stable disease; PR, partial response; CR, complete response; LCR, local control rate.

### Local control rate and Overall survival by treatment modalities

**Figure 4:**
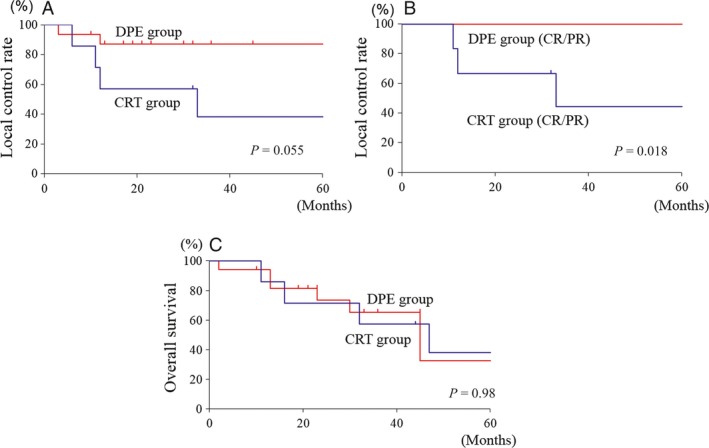
Local Control Rate and Overall Survival by the DPE and CRT groups. (A) The DPE group had a better 3‐year LCR compared with the CRT group. (B) In patients who had a good response to chemotherapy, the DPE group had a significantly better 3‐year LCR compared with the CRT group. (C) The treatment modalities were not associated with OS. CRT, chemoradiotherapy; DPE, delayed primary excision; LCR, local control rate.

As a local control, the DPE group had a better 3‐year LCR compared with the CRT group (DPE group vs. CRT group, 87% vs. 38%, *P *=* *0.055). Particularly, in the patients with a good response to chemotherapy (CR, PR), the DPE group had a significantly better 3‐year LCR compared with the CRT group (DPE group vs. CRT group, 100% vs. 44%, *P *=* *0.018). The treatment modalities were not associated with the 3‐year OS (DPE group vs. CRT group, 65% vs. 57%: *P *=* *0.98) (Fig. 4).

## Discussion

Pediatric RMS is very sensitive to chemotherapy and radiotherapy. With the modern treatments of IC followed by concurrent chemoradiotherapy, the survival rates have improved from 30% to 70% 5. There is no doubt about the significance of chemotherapy. On the other hand, adult RMS is more aggressive and has a higher overall mortality rate than pediatric RMS 2.

In this study, we chose a more intensive local treatment including surgery for the following four reasons: (1) adult RMS has a prognosis worse than that of pediatric RMS, (2) the intensity of chemotherapy is decreased in adult cases because of increased sensitivity to hematologic toxicity, (3) the poorer response to chemotherapy for adult RMS than for pediatric RMS, and (4) the lower tendency for deformity in adults than in pediatric cases because of skeletal maturity and plastic surgical reconstruction.

In general, initial surgery was recommended in the COG guidelines 6; however, the European Pediatric Soft Tissue Sarcoma Study Group recommended a complete surgical resection after IC for head and neck RMS. The timing and choice of surgical procedure has been controversial.

Moreover, no prospective study has been conducted to assess the significance of surgery after IC. In the treatment strategy of RMS, surgery after IC was also called DPE 7. D9803 study encouraged the use of DPE for selected tumors that were initially unresected and evaluated the use of dose‐reduced RT after DPE 7. Only specific anatomic sites were considered to be candidates for DPE, including the extremity, bladder, and trunk.

In many adult head and neck RMSs, the initial complete surgical resection may be difficult because of the anatomical limitations. Therefore, we performed the surgical resection after IC. The extent of resection was defined by the response to chemotherapy.

In our analysis, after surgery, no patients had gross residual tumors and 59% of the patients had complete resection with negative surgical margins. There had been no report about surgical outcomes after DPE in head and neck RMS, but these results had almost same outcomes as that of other anatomical sites such as bladder or trunk. (bladder: complete resection by DPE 45%; trunk: complete resection by DPE 45%) 6, 7.

In this analysis, patients who had a complete resection did not have a local recurrence. On the other hand, both patients who had a local recurrence also had microscopic residual tumors and a poor response to IC. Moreover, the poor response to IC was significantly associated with a worse 3‐year LCR. Conversely, there was no local recurrence in the patients who had a good response to IC, even with the presence of microscopic residual tumors. In previous studies, the correlation between outcome and imaging response after IC has been controversial 8, 9, 10. Adequate margins for resection have been a major concern to head and neck surgeons, because of the anatomical limitations. In any situation, a complete surgical resection was recommended. Especially in the cases with poor responses to IC, radical resection with wide surgical margins was required for local disease control. On the other hand, in cases with good response to IC, conservative resection with close surgical margins might be acceptable for organ preservation.

Pathologically, 82% of patients had viable tumors after IC; moreover, among the patients who had clinical CR, 33% had viable tumors. This was consistent with a previous report, where 59–79% of tumors contained viable tumor after IC 6, 11. Patients with pathological CR after IC did not have a local recurrence and had a better 3‐year LCR (viable tumor in DPE specimen, positive vs. negative: 85% vs. 100%, *P *=* *0.53). However, even with the presence of viable tumors in DPE specimens, there was no local recurrence in the patients who underwent complete surgical resection or achieved good response to IC. In addition, among patients with good response to the IC, the DPE group had a significantly better local control rate than the CRT group. These results suggest that DPE may impact local control of adult RMS, even with good response to chemotherapy. However, this analysis was a nonrandomized retrospective study, and there was an inherent bias in patient selections, namely CRT group tended to have more locally advanced patients. Further studies are needed to clarify the role of DPE.

ARST0531 study recommended an earlier start for RT for patients with meningeal impingement 12. The rationale for the early RT was the lower local failure rate for patients with meningeal impingement who had received RT within 2 weeks of starting systemic therapy (18% vs. 33% for a delay beyond 2 weeks). With the addition of surgery after IC, the timing of radiotherapy was delayed. However, in many patients, more intensive local treatment was implemented by the margin‐negative skull base surgery, and postoperative chemoradiotherapy was possible within an average of 3 weeks after surgery. Therefore, the surgery might be useful for the local treatment of parameningeal adult RMS. We recommend performing the surgery prior to the radiotherapy because otherwise the risk of postoperative complications, such as liquorrhea or poor wound healing, will increase.

The indication for orbital preservation was unclear. Generally, orbital exenteration should be avoided as a first treatment. However, in this study, half of the patients with positive periorbital margins had local recurrence and were difficult to salvage. Further studies will be needed to identify the rationales for orbital exenteration.

For local control, the DPE group had a better 3‐year LCR compared with the CRT group; however, there was no impact on overall survival, because of the distant recurrence. The long cessation of chemotherapy by surgery might become a distant recurrence. In our analysis, the median cessation duration was 24 days (7–63 days). In this study, for short cessation of chemotherapy, conservative surgery such as partial maxillectomy was performed in patients with good response to IC. In these patients, immediate resumption of chemotherapy (about 7 days) was possible because of minimally invasive surgery. However, in patients with poor response to IC, radical surgery, such as skull base surgery or resection with free flap reconstruction, was performed. Such highly invasive surgeries needed postoperative rehabilitation; thus, a few weeks were required before chemotherapy could be resumed (14–24 days). However, long cessation duration did not affect the 3‐year distant control rate (>21 days vs. <21 days; 71% vs. 60% *P* = 0.92). Also, the response to IC did not affect the 3‐year distant control rate (SD/PD vs. PR/CR, 75% vs. 69%; *P* = 0.84). To prevent distant recurrences, further improvement of the chemotherapy regimens will be required.

## Conclusions

DPE may impact local control of localized nonmetastatic adult head and neck RMS. Poor response to IC was a risk factor for local recurrence. However, this is a small‐sample nonrandomized retrospective study, and further studies are needed to clarify the role of surgery. To prevent distant metastases, a more effective chemotherapy regimen will be required.

## Conflict of Interest

The authors report no conflicts of interest.
